# The relationship between ambulatory arterial stiffness index and left ventricular diastolic dysfunction in HFpEF: a prospective observational study

**DOI:** 10.1186/s12872-022-02679-6

**Published:** 2022-06-02

**Authors:** Hanwen Zhang, Weiwei Hu, Yu Wang, Jie Liu, Linna You, Qian Dong, Guanglei Chang, Xiaocheng Cheng, Zhiqiang Liu, Dongying Zhang

**Affiliations:** Department of Cardiology, The First Affiliated Hospital of Chongqing Medical University, No.1 Youyi Street, Yuzhong District, Chongqing, 400016 China

**Keywords:** Ambulatory arterial stiffness index, Diastolic dysfunction, Heart failure with preserved ejection fraction

## Abstract

**Background:**

The relationship between ambulatory arterial stiffness index (AASI) and left ventricular diastolic dysfunction (LVDD) in patients with heart failure with preserved ejection fraction (HFpEF) is unknown. We aimed to investigate the association between the AASI and LVDD in HFpEF.

**Methods:**

We prospective enrolled consecutive patients with HFpEF in Chongqing, China. Twenty-four-hour ambulatory blood pressure monitoring (24 h-ABPM) and echocardiography were performed in each patient. AASI was obtained through individual 24 h-ABPM. The relationship between AASI and LVDD was analyzed.

**Results:**

A total of 107 patients with HFpEF were included. The mean age was 68.45 ± 14.02 years and 63 (59%) were women. The patients were divided into two groups according to the upper normal border of AASI (0.55). AASI > 0.55 group were more likely to be older, to have higher mean systolic blood pressure and worsen left ventricular diastolic function than AASI group ≤ 0.55. AASI was closely positive related to the diastolic function parameters, including mean E/e′ (r = 0.307, *P* = 0.001), septal E/e′ (r = 0.290, *P* = 0.002), lateral E/e′ (r = 0.276, *P* = 0.004) and E (r = 0.274, *P* = 0.004). After adjusting for conventional risk factors, AASI was still an independent risk factors of mean E/e′ > 10 in patients with HFpEF (OR: 2.929, 95%CI: 1.214–7.064, *P* = 0.017), and the association between AASI and mean E/e′ > 14 was reduced (OR: 2.457, 95%CI: 1.030–5.860, *P* = 0.043). AASI had a partial predictive value for mean E/e′ > 10 (AUC = 0.691, *P* = 0.002), while the predictive value for mean E/e′ > 14 was attenuated (AUC = 0.624, *P* = 0.034).

**Conclusion:**

AASI was positive related to E/e′ in HFpEF and might be an independent risk factor for the increase of mean E/e′.

**Supplementary Information:**

The online version contains supplementary material available at 10.1186/s12872-022-02679-6.

## Introduction

Ambulatory arterial stiffness index (AASI) is defined as 1 minus the regression slope of diastolic on systolic blood pressure (BP) values obtained from the 24-h ambulatory blood pressure monitoring (ABPM) recordings [1, 2]. It has been proposed as a novel indicator of arterial stiffness and has the advantages by the low cost and noninvasive. Substantial reports revealed that increased arterial stiffness was associated with preclinical target organ damage and increased risk of cardiovascular mortality and morbidity in hypertension [3–5].

Heart failure with preserved ejection fraction (HFpEF) is characterized by left ventricular diastolic dysfunction (LVDD) and cardiac remodeling (fibrosis, inflammation, and hypertrophy), which has become a major cause of hospitalization for the elders [6–8]. Hypertension, diabetes and obesity were risk factors for HFpEF and were associated with arterial stiffness raise [9, 10]. It was reported that microvascular dysfunction and chronic low-grade inflammation have been proposed to participate in HFpEF development [11]. Other parameters reflecting arterial stiffness such as cardio-ankle vascular index (CAVI), have been reported associated with the hospitalization of HFpEF patients [12], but the role of AASI in HFpEF is still unknown. The objective of the present study was to investigate the relationship between ambulatory arterial stiffness index and left ventricular diastolic dysfunction in patients with HFpEF.

## Methods

### Study design and participants

**Fig. 1:**
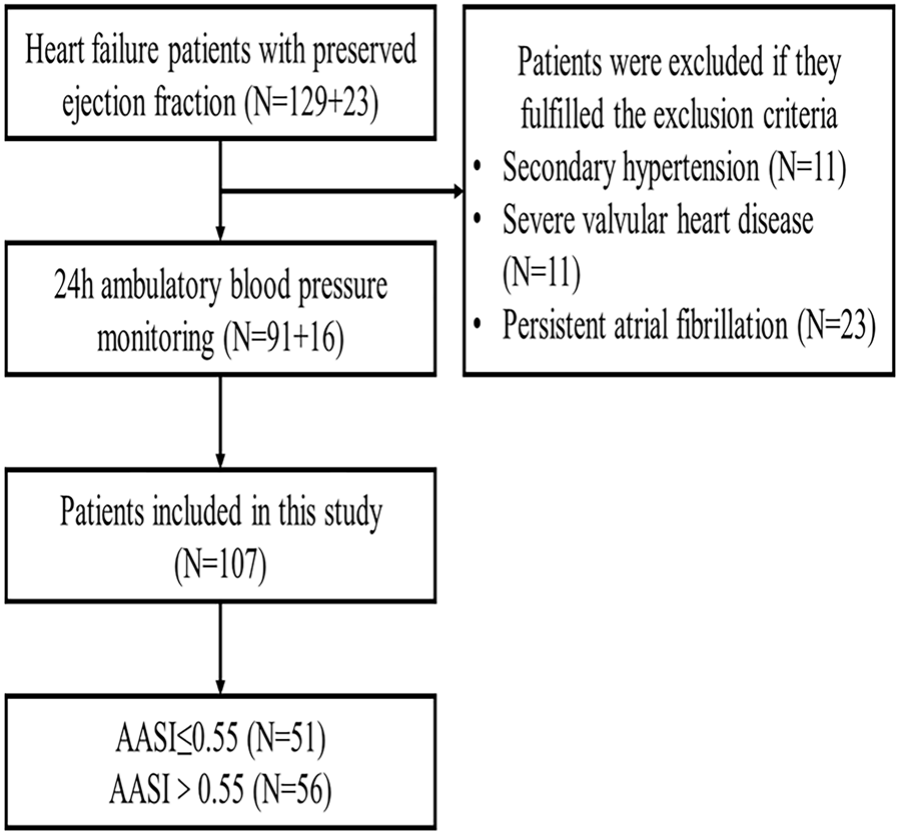
Flow diagram for the study population

From November 2020 to February 2021, we conducted a prospective observational study registry with clinicaltrials.gov identifier NCT05059769. This study initially enrolled 129 patients with HFpEF in the First Affiliated Hospital of Chongqing Medical University and we added 23 patients from March 10 to 24, 2022. Inclusion criteria included age > 18 years and conform to HFpEF diagnostic criteria (Left ventricular ejection fraction ≥ 50%, typical symptoms and signs of heart failure, HFA-PEFF score ≥ 5) [13], whereas the exclusion criteria were secondary hypertension (N = 11), severe valvular heart disease (N = 11) and persistent atrial fibrillation (N = 23). Finally, a total of 107 patients with HFpEF were included (Fig. 1). Informed consent was obtained from the patients, and the study was approved by the institutional ethics board of the First Affiliated Hospital of Chongqing Medical University (approval NO.2020-606). Baseline clinical and demographic information was obtained from all patients. Body mass index (BMI) was calculated as weight (kg)/height (m^2^). 24-h ABPM and echocardiography were carried out on all patients during hospitalization.

### Diagnosis of HFpEF

Echocardiography was performed in patients with suspected HFpEF (Left ventricular ejection fraction ≥ 50%, typical symptoms and signs of heart failure). HFA-PEFF score was calculated by left ventricular diastolic function index and NT-proBNP. Score ≥ 5 was considered to be diagnostic of HFpEF [13].

### 24 h-ABPM

Twenty-four-hour ABPM was performed using the Mobil-O-Graph NG (Z02505), a non-invasive ambulatory BP monitoring instrument. BP readings were obtained at 15-min intervals during the day and at 30-min intervals during the night. Of the total readings, ≥ 80% was considered valid. Furthermore, for the records valuable, at least 14 measurements during the daytime period or at least 7 measurements during the night or rest period were required [14].

### Ambulatory arterial stiffness index (AASI)

AASI is defined as 1 minus the regression slope of diastolic on systolic blood pressure values obtained from the 24 h-ABPM recordings [1, 2]. AASI was obtained as follows:

$${\text{AASI }} = { 1} - {\text{slope }}\left( {{\text{diastolic BP}}/{\text{systolic BP}}} \right)$$

### Echocardiography

The cardiac diastolic function of HFpEF was assessed by transthoracic echocardiography (Vivid E95, AU11403, GE Vingmed Ultrasound AS). Using the parasternal short-axis two-dimensional view to image the heart and record an M-mode echocardiogram at the level of the papillary muscles. Cardiac function parameters, such as left ventricular ejection fraction (LVEF), diastolic interventricular septum thickness (IVSd), diastolic left ventricular posterior wall thickness (LVPWd), left atrium volume index (LAVI), left ventricle mass index (LVMI), the peak velocity of the filling peak in the early diastolic period (E), the peak velocity of the filling peak in the late diastolic period (A), the E/A ratio (E/A), septal mitral annular early diastolic peak velocities (Septal e′), lateral mitral annular early diastolic peak velocities (Lateral e′), the ratio of the early diastolic transmitral filling velocity to the early diastolic septal tissue velocity (Septal E/e′) and the ratio of early diastolic transmitral flow velocity to the mitral annular velocity at the lateral wall (Lateral E/e′) were measured by the same investigator. At the same time, tricuspid annular plane systolic excursion (TAPSE) and plane contraction offset velocity of tricuspid annulus (TAPSE-S) reflecting right ventricular function were measured as well [15].

### Data collection

Data on epidemiological information, medical history, exposure history, underlying comorbidities, symptoms, signs, laboratory, and radiological characteristics were obtained from electronic medical records. All the data were collected by two investigators independently and double-checked by other investigators.

### Definition

The patients would be divided into two groups according to the upper normal border of AASI = 0.55 [1, 2]. In the logistic regression analysis, mean E/e′ > 10 and 14 were chose to explore the association between AASI and LVDD.

### Statistical analysis

Categorical variables were described as frequency rates and percentages, and continuous measurements as mean (standard deviation: [SD]) if they are normally distributed or median (interquartile range: [IQR]) if they are not. X^2^ test was used to test for differences in categorical variables among the two groups. T test or Mann–Whitney test was used to compare continuous variables according to the normal distribution or not. Pearson correlation analysis was used for assessing the correlates of left ventricular diastolic function. Multivariate linear regression was used to test whether AASI was independently correlated with mean E/e′. logistic regression analysis was used to test independent factors of LVDD. ROC curve for AASI to predict LVDD was performed to further reveal the association between AASI and LVDD. All P values were two-tailed, and significance was set at *P* < 0.05. Statistical analyses were performed using SPSS software (version 22.0).

## Results

### Baseline characteristics

The study initially enrolled 129 patients with HFpEF and we added 23 patients from March 10 to 24, 2022. Patients who had secondary hypertension (n = 11), severe valvular heart disease (n = 11) and persistent atrial fibrillation (n = 23) were excluded (Fig. 1). Finally, a total of 107 HFpEF patients were included and the average HFA-PEFF score was 5.51 ± 0.50. Table 1 showed the baseline clinical and demographic data in total group and two subgroups. The mean age of the patients was years and 63 (59%) were women. More than half had comorbidities including hypertension (74, 69%), diabetes (36, 34%), coronary artery disease (CAD) (53, 50%) and chronic obstructive pulmonary disease (COPD) (13, 12%). The number of patients who were taking antihypertensive drugs was shown as follows: ARB (45, 42%), CCB (36, 34%), beta-blockers (45, 42%), diuretics (60, 56%). Median plasma NT-proBNP was 840 (IQR 310–1748) pg/ml. Based on NYHA classification, classes II and III–IV were 34 (32%) and 73 (68%), respectively. Echocardiographic results showed that patients had normal left ventricular systolic function and impaired diastolic function. The average systolic and diastolic blood pressure were 122.87 ± 17.34 and 69.71 ± 9.30 mmHg, respectively. AASI was calculated from ambulatory blood pressure data and the average value was 0.55 ± 0.19 which was close to the upper normal border (0.55). The patients were divided into two groups (AASI ≤ 0.55, AASI > 0.55). The AASI > 0.55 group showed higher age, ave-systolic blood pressure and worsen left ventricular diastolic function. Age, ave-systolic BP, E, septal E/e′, lateral E/e′ and mean E/e′ in the AASI > 0.55 group were significantly higher than in the AASI group ≤ 0.55, while septal e′ was lower. The incidence rate of mean E/e′ > 10 was marked higher in AASI > 0.55 group than AASI ≤ 0.55 group (80% vs 55%). There was no significant difference in the other clinical parameters between the two groups, including comorbidities, height, weight, BMI, ave-dBP, NYHA class, NT-proBNP, LVEF, LAVI and LVMI. We also focused on the right ventricular systolic function, in which the TAPSE and TAPSE-S did not differ between the two groups.

**Table 1:** Baseline clinical characteristics of included patients with HFpEF

Characteristics	All (N = 107)	AASI ≤ 0.55 (N = 51)	AASI > 0.55 (N = 56)	*P*
Age (years)	68.45 ± 14.02	63.41 ± 15.08	73.04 ± 11.29	< 0.001
Female	63 (59%)	29 (57%)	34 (61%)	0.686
*History*				
Hypertension	74 (69%)	31 (61%)	43 (77%)	0.074
Diabetes	36 (34%)	13 (25%)	23 (41%)	0.088
CAD	53 (50%)	21 (41%)	32 (57%)	0.099
COPD	13 (12%)	6 (12%)	7 (13%)	0.907
Smoking	19 (18%)	7 (14%)	12 (21%)	0.298
Drinking	26 (24%)	12 (24%)	14 (25%)	0.859
*Medication*				
ARB	45 (42%)	14 (27%)	31 (55%)	0.004
CCB	36 (34%)	16 (31%)	20 (36%)	0.635
Beta-blockers	45 (42%)	18 (35%)	27 (48%)	0.176
Diuretics	60 (56%)	29 (57%)	31 (55%)	0.876
*GM*				
Height (m)	1.60 ± 0.09	1.60 ± 0.09	1.59 ± 0.09	0.663
Weight (kg)	61.00 ± 13.56	62.34 ± 16.58	59.77 ± 10.07	0.330
BMI (kg/m^2^)	23.81 ± 3.83	24.18 ± 4.65	23.48 ± 2.88	0.349
Ave-sBP (mmHg)	122.87 ± 17.34	118.43 ± 17.38	126.91 ± 16.43	0.011
Ave-dBP (mmHg)	69.71 ± 9.30	70.69 ± 9.34	68.82 ± 9.26	0.303
AASI	0.55 ± 0.19	0.39 ± 0.14	0.69 ± 0.10	< 0.001
*NYHA classification*				
II	34 (32%)	16 (31%)	18 (32%)	0.932
III–IV	73 (68%)	35 (69%)	38 (68%)	0.932
HFA-PEFF score	5.51 ± 0.50	5.49 ± 0.50	5.54 ± 0.50	0.642
Mean E/e’ > 10	73 (68%)	28 (55%)	45 (80%)	0.005
NT-proBNP (pg/mL)	840 (310–1748)	919 (310–2192)	818 (308–1481)	0.405
*Echo data*				
IVSd (mm)	11.21 ± 2.17	11.06 ± 1.94	11.34 ± 2.37	0.507
LVPWd (mm)	10.64 ± 1.51	10.73 ± 1.67	10.55 ± 1.35	0.558
LAVI (ml/m^2^)	38.34 ± 17.18	35.24 ± 16.46	41.17 ± 17.47	0.074
LVMI (g/m^2^)	119.642 ± 34.29	118.30 ± 27.55	120.86 ± 39.65	0.702
LVEF (%)	61.80 ± 5.43	62.20 ± 6.01	61.45 ± 4.87	0.478
E (cm/s)	71.30 ± 23.40	65.25 ± 19.71	76.81 ± 25.25	0.010
E/A	0.83 ± 0.33	0.83 ± 0.33	0.84 ± 0.33	0.850
Septal e′ (cm/s)	5.24 ± 2.01	5.68 ± 2.24	4.85 ± 1.70	0.032
Lateral e′ (cm/s)	6.78 ± 1.99	7.13 ± 2.00	6.46 ± 1.94	0.082
Septal E/e′	15.02 ± 6.31	13.03 ± 5.76	16.82 ± 6.28	0.002
Lateral E/e′	11.48 ± 5.58	9.67 ± 3.12	13.13 ± 6.73	< 0.001
Mean E/e′	13.25 ± 5.48	11.35 ± 4.06	14.98 ± 6.05	0.001
TAPSE (mm)	18.98 ± 3.74	18.64 ± 3.90	19.29 ± 3.59	0.382
TAPSE-S (cm/s)	11.69 ± 2.80	11.78 ± 3.19	11.62 ± 2.42	0.771

*AASI* ambulatory arterial stiffness index, *ARB* angiotensin receptor blockers, *Ave-sBP* averaged systolic blood pressure, *Ave-dBP* averaged diastolic blood pressure, *BMI* body mass index, *CAD* coronary artery disease, *CCB* calcium channel blockers, *COPD* chronic obstructive pulmonary disease, *E* the peak velocity of the filling peak in the early diastolic period, *E/A* the E/A ratio, *GM* general measurement, *HFpEF* heart failure with preserved ejection fraction, *HFA-PEFF* score ≥ 5 is considered to be diagnostic of HFpEF, while score ≤ 1 is considered to make a diagnosis of HFpEF very unlikely, *IVSd* ventricular septal end diastolic thickness, *LAVI* left atrium volume index, *LVEF* left ventricular ejection fraction, *Lateral e*′ lateral mitral annular early diastolic peak velocities, *LVMI* left ventricle mass index, *LVPWd* left ventricular posterior wall end diastolic thickness, Lateral E/e′, the ratio of early diastolic transmitral flow velocity to mitral annular velocity at the lateral wall; Mean E/e′, average septal-lateral E/e′ ratio; *NT-proBNP* N-terminal pro-B-type natriuretic peptide, *NYHA* New York heart association, *Septal e’* septal mitral annular early diastolic peak velocities, Septal E/e′, the ratio of the early diastolic transmitral filling velocity to the early diastolic septal tissue velocity, *TAPSE* tricuspid annular plane systolic excursion, *TAPSE-S* tricuspid annular plane systolic excursion velocity

### Pearson correlations between clinical characteristics and left ventricular diastolic function

**Fig. 2:**
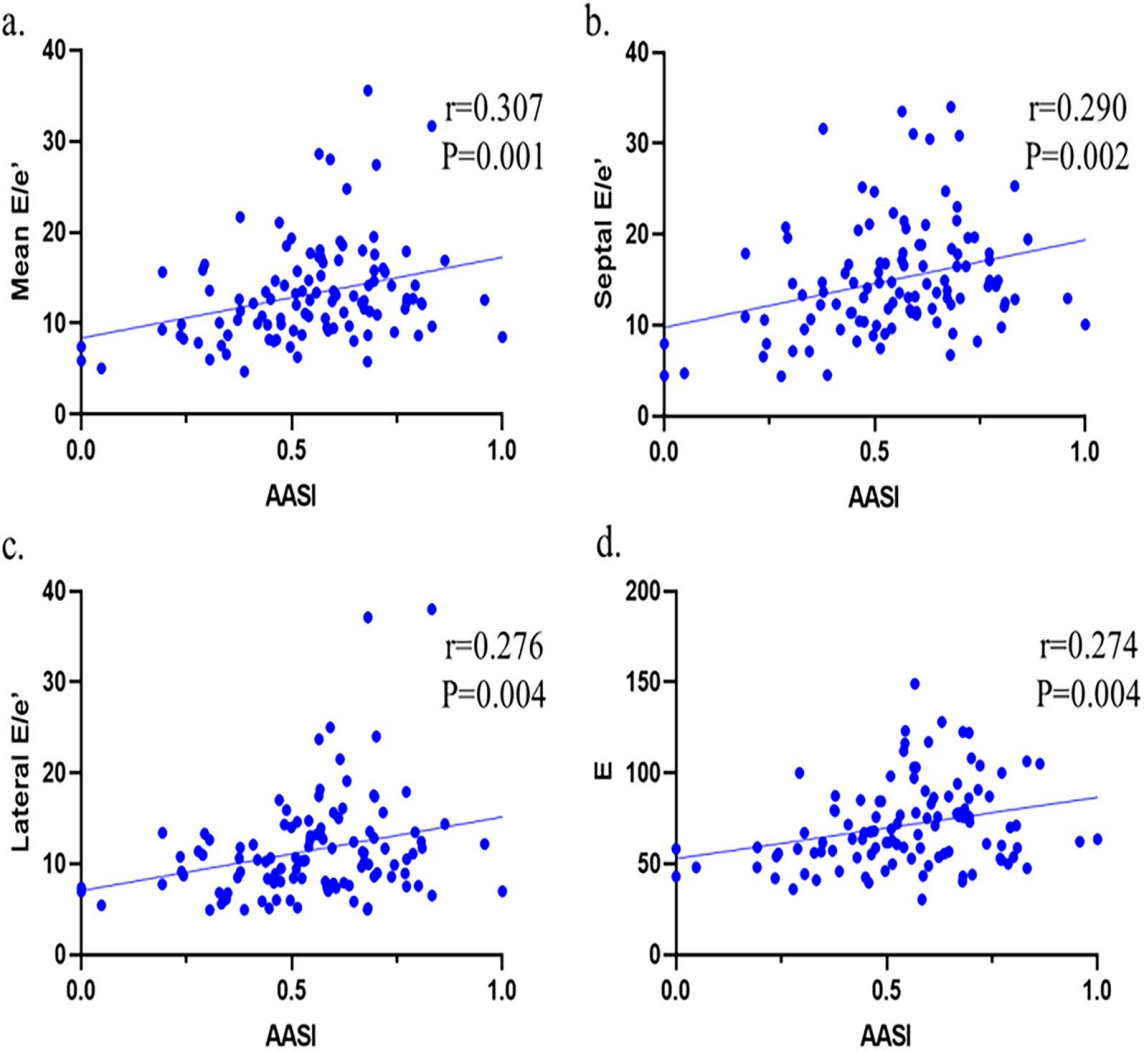
Correlation of the AASI with left ventricular diastolic function

The relationships for AASI and other clinical parameters between left ventricular diastolic function, including E, septal E/e′, lateral E/e′ and mean E/e′ were conducted by Pearson correlations analysis. The results were shown in Table 2. We found that AASI was closely positive related to the diastolic function parameters, including mean E/e′ (r = 0.307, *P* = 0.001; Fig. 2a), septal E/e′ (r = 0.290, *P* = 0.002; Fig. 2b), lateral E/e′ (r = 0.276, *P* = 0.004; Fig. 2c) and E (r = 0.274, *P* = 0.004; Fig. 2d). Age and ave-sBP were also related to mean E/e’. We studied the relationships for AASI between right ventricular systolic function, and Pearson correlation analysis indicated that AASI was related to TAPSE (r = 0.203, *P* = 0.040) (Additional file 1: Table S1). The results of the correlation analysis showed that AASI had the highest correlation with mean E/e′, so we took mean E/e′ as the main index to evaluate left ventricular diastolic function in the follow-up analysis.

**Table 2:** Pearson correlations between clinical characteristics and left ventricular diastolic function

parameters	Mean E/e′		Septal E/e′		Lateral E/e′		E	
	*r*	*P*	*r*	*P*	*r*	*P*	*r*	*P*
Age	0.215	0.026	0.231	0.017	0.161	0.099	0.201	0.038
Height	− 0.085	0.383	− 0.140	0.151	− 0.009	0.923	− 0.063	0.522
Weight	− 0.063	0.521	− 0.118	0.225	0.010	0.915	− 0.112	0.252
BMI	− 0.023	0.815	− 0.060	0.540	0.023	0.817	− 0.087	0.373
Ave-sBP	0.272	0.005	0.242	0.012	0.261	0.007	0.141	0.147
Ave-dBP	− 0.055	0.570	− 0.063	0.520	− 0.038	0.698	− 0.111	0.257
AASI	0.307	0.001	0.290	0.002	0.276	0.004	0.274	0.004

*AASI* ambulatory arterial stiffness index, *Ave-sBP* averaged systolic blood pressure, *Ave-dBP* averaged diastolic blood pressure, *BMI* body mass index, E, the peak velocity of the filling peak in the early diastolic period; Lateral E/e′, The ratio of early diastolic transmitral flow velocity to mitral annular velocity at the lateral wall; Mean E/e′, Average septal-lateral E/e′ ratio; Septal E/e′, The ratio of the early diastolic transmitral filling velocity to the early diastolic septal tissue velocity

### Multivariate linear regression analysis

To investigate the independent relevant factors of mean E/e′, we added age, ave-sBP and AASI into the multivariate linear regression analysis Model. In Model I, after adjusting age, AASI was independently relevant with mean E/e′ (β: 0.264, *P* = 0.012). In Model II, after adjusting ave-sBP, AASI was independently relevant with mean E/e′ (β: 0.244, *P* = 0.013). The results of Model III showed that age (β: 0.080, *P* = 0.439) and ave-sBP (β: 0.185, *P* = 0.061) were not independently associated with mean E/e′, but AASI was still an independent relevant factor of mean E/e′ (β: 0.211, *P* = 0.049) (Table 3).

**Table 3:** Multivariate linear regression analysis

parameters	Mean E/e′				
	B	SE	β	t	*P*
*Model I*					
Constant	6.493	2.569		2.527	0.013
Age	0.038	0.041	0.097	0.936	0.352
AASI	7.612	2.993	0.264	2.543	0.012
*Model II*					
Constant	1.955	3.606		0.542	0.589
Ave-sBP	0.061	0.031	0.192	1.976	0.051
AASI	7.045	2.798	0.244	2.518	0.013
*Model III*					
Constant	0.601	4.011		0.150	0.881
Age	0.031	0.040	0.080	0.777	0.439
Ave-sBP	0.058	0.031	0.185	1.897	0.061
AASI	6.081	3.065	0.211	1.984	0.049

*AASI* ambulatory arterial stiffness index, *Ave-sBP* averaged systolic blood pressure, Mean E/e′, Average septal-lateral E/e’ ratio

Model I: R = 0.319, R^2^ = 0.102, Adjusted R^2^ = 0.085, F = 5.898, *P* = 0.004

Model II: R = 0.357, R^2^ = 0.127, Adjusted R^2^ = 0.110, F = 7.571, *P* = 0.001

Model III: R = 0.364, R^2^ = 0.132, Adjusted R^2^ = 0.107, F = 5.229, *P* = 0.002

### Logistic regression demonstrating the risk factors of left ventricular diastolic dysfunction

We chose mean E/e′ > 10 as the risk threshold for LVDD in Table 4. Univariate regression analysis showed that AASI was associated with increased risk of mean E/e′ > 10 [Odds ratio: 3.360, 95% Confidence interval (CI) 1.423–7.937, *P* = 0.006]. Multivariate regression analysis showed that after adjusting for conventional risk factors including ave-sBP and CAD, AASI was still an independent risk factor (OR: 2.929, 95% CI 1.214–7.064, *P* = 0.017) (Table 4).

**Table 4:** Logistic regression of AASI > 0.55 predicting mean E/e’ > 10

Risk factors	Mean E/e′ > 10					
	Univariate			Multivariate		
	OR	95%CI	*P*	OR	95%CI	*P*
AASI > 0.55	3.360	1.423–7.937	0.006	2.929	1.214–7.064	0.017
Age > 65	1.623	0.696–3.783	0.262			
Ave-sBP > 135	2.830	0.884–9.057	0.080	2.295	0.682–7.721	0.180
BMI > 25	1.630	0.664–4.002	0.286			
Female	1.704	0.748–3.881	0.205			
Hypertension	1.989	0.842–4.702	0.117			
Diabetes	1.630	0.664–4.002	0.286			
CAD	2.349	1.012–5.450	0.047	1.981	0.822–4.771	0.128
COPD	1.640	0.421–6.390	0.476			

*AASI* ambulatory arterial stiffness index, *Ave-sBP* averaged systolic blood pressure, *BMI* body mass index, *CAD* coronary atherosclerotic heart disease, *COPD* chronic obstructive pulmonary disease, Mean E/e′, Average septal-lateral E/e′ ratio

Mean E/e′ > 14 was also chose as the risk threshold for LVDD in Additional file 1: Table S2. Univariate regression analysis showed that AASI was associated with increased risk of E/e′ > 14 (OR: 2.817, 95% CI 1.224–6.481, *P* = 0.015). Multivariate regression analysis showed that after adjusting for conventional risk factors including female, diabetes, and CAD, AASI was still an independent risk factor (OR: 2.457, 95% CI 1.031–5.860, *P* = 0.043) (Additional file 1: Table S2).

### ROC curve for AASI to predict left ventricular diastolic dysfunction

**Fig. 3:**
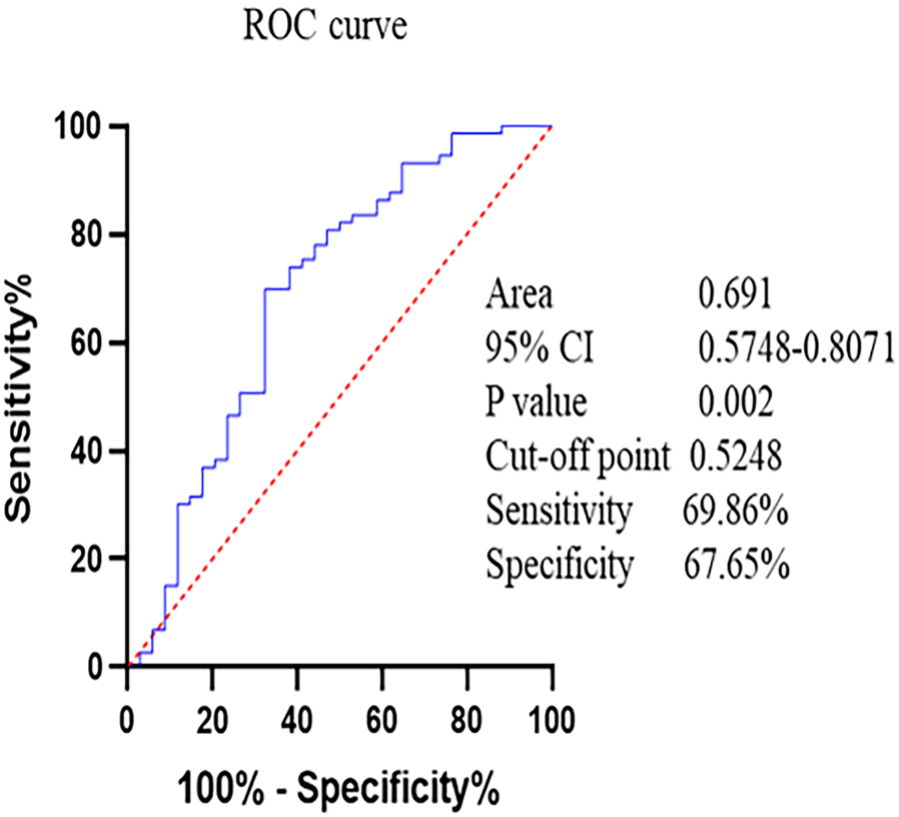
ROC curve for AASI to predict mean E/e′ > 10

We performed the ROC curve analysis of AASI predicting mean E/e′ > 10 and 14, respectively. AASI had a better predictive value for mean E/e′ > 10 in patients with HFpEF (AUC = 0.691, *P* = 0.002, Fig. 3), We found the cut-off point by the Jordan index (the sum of sensitivity and specificity minus 1). AASI > 0.5248 was the cut-off point with sensitivity and specificity values of 69.86% and 67.65%, respectively. While the predictive value for mean E/e′ > 14 was reduced (AUC = 0.624, *P* = 0.034, Additional file 1: Fig. S1). AASI > 0.5401 was the cut-off point with sensitivity and specificity values of 73.68% and 55.07%, respectively.

## Discussion

The major findings of this study were the following: (1) HFpEF with high AASI were more likely to be older, to have higher mean systolic blood pressure and worsen left ventricular diastolic function; (2) AASI was positively correlated with left ventricular diastolic dysfunction parameter (E/e′); (3) AASI might be an independent risk factor for the increase of mean E/e′ in patients with HFpEF.

HFpEF is a group of syndromes with left ventricular diastolic dysfunction as the main clinical manifestation, often accompanied by risk factors such as advanced age, hypertension and diabetes [16, 17], which was consistent with the characteristics of our cohort study. AASI was determined from the records of ABPM has been proposed as a surrogate indicator of arterial stiffness [1, 2]. Several studies reported that AASI may be related to diastolic function and prognosis in hypertension and diabetes [3, 4, 18]. Nevertheless, the role of AASI in patients with HFpEF has not been reported.

In the present study, we found that HFpEF with high AASI were more likely to be older, to have higher systolic blood pressure. This can be explained by the reason that the elderly and high sBP were major factors contributed to arterial stiffness [19, 20]. The septal E/e′, lateral E/e′ and mean E/e′ are representative parameters that reflect left ventricular diastolic dysfunction [21]. As we expected, above mentioned parameters were significantly higher in the AASI > 0.55 group than in the AASI ≤ 0.55 group. Pearson correlation also indicated that AASI was closely positive related to the parameters of diastolic dysfunction. Although the E/A value is often regarded as a parameter of diastolic dysfunction, we did not observe the correlation between AASI and E/A in HFpEF. The possible reason was that E/A may have similar values in different stages of diastolic dysfunction [22].

In our study, Pearson correlation analysis confirmed that the age and ave-sBP were related to E/e′. However, after adjusting for above mentioned risk factors, an independent correlation between AASI and mean E/e′ was still observed in the multivariate linear regression analysis.

Previous studies reported that conventional risk factors such as age, hypertension, diabetes, BMI and so on were closely positive related to cardiac dysfunction [23–25]. E/e′ > 14 was one of the common indicators for the diagnosis of LVDD [26]. In different studies, the authors chose different E/e′ values to explore the relationship between E/e′ and LVDD [27, 28]. In the logistic regression analysis, we chose both mean E/e′ > 10 and 14 to explore the relationship between AASI and LVDD. Univariate logistic regression analysis found that AASI > 0.55, ave-sBP > 135 and CAD were associated with an increased risk of mean E/e′ > 10. While AASI > 0.55, female, diabetes and CAD were associated with an increased risk of mean E/e′ > 14. After adjusting for the above factors, AASI was still an independent risk factors of mean E/e′ > 10 and 14, respectively. Additionally, we also focused on the right ventricular systolic function, in which the TAPSE and TAPSE-S did not differ between the two groups, indicating that AASI might be a risk factor for left ventricular diastolic dysfunction rather than right ventricular in patients with HFpEF.

ROC curve analysis indicated that AASI might have a predictive value for mean E/e′ > 10 in patients with HFpEF, while the predictive value for mean E/e′ > 14 was attenuated. AASI is one of the major indicators of arterial stiffness. AASI comes from 24-h ambulatory blood pressure monitoring data and is affected by the dynamic changes of blood pressure. Its measurement is different from the pulse wave pulse speed while it is closely correlated with aortic pulse wave velocity and the central and peripheral systolic augmentation indexes. Our findings supported that arterial stiffness might serve as risk factors for the development of HFpEF [29, 30]. Severe diastolic dysfunction was associated with an increased risk of major adverse cardiovascular event [31]. Therefore, AASI might be a predictor of adverse events in patients with HFpEF, which needs to be confirmed in the future.

The present study has several limitations that should be considered. First, because this is an observational study, we cannot determine the causality of the results of the study. Second, the sample of this study was small and our findings still need to be further confirmed. Third, because of the small sample, whether AASI was associated with major cardiovascular adverse events in HFpEF could not be determined. Fourth, E/e’ was only one of the indicators chose in our cohort to reflect LVDD, while other indicators should be involved in further study. We found the predicting value of AASI for LVDD was limited, whether it had the predicting value for LVDD needs more research.

## Conclusion

AASI was positive related to E/e′ in HFpEF and might be an independent risk factor for the increase of mean E/e′.

## Supplementary Information

**Additional file 1. Table S1**. Pearson correlations between clinical characteristics and right ventricular systolic function. **Table S2**. Logistic regression of AASI > 0.55 predicting mean E/e′ > 14. **Figure S1**. ROC curve for AASI to predict mean E/e′ > 14.

## Data Availability

The data that support the findings of this study are available from the corresponding author upon reasonable request.

## Abbreviations

AASI: Ambulatory arterial stiffness index
ARB: Angiotensin receptor blockers
Ave-sBP: Averaged systolic blood pressure
Ave-dBP: Averaged diastolic blood pressure
BMI: Body mass index
CAD: Coronary artery disease
CAVI: Cardio-ankle vascular index
CCB: Calcium channel blockers
COPD: Chronic obstructive pulmonary disease
E: The peak velocity of the filling peak in the early diastolic period
E/A: The E/A ratio
HFpEF: Heart failure with preserved ejection fraction
HFA-PEFF: Score ≥ 5 is considered to be diagnostic of HFpEF, while score ≤ 1 is considered to make a diagnosis of HFpEF very unlikely
GM: General measurement
IVSd: Ventricular septal end diastolic thickness
LAVI: Left atrium volume index
LVDD: Left ventricular diastolic dysfunction
LVEF: Left ventricular ejection fraction
LVMI: Left ventricle mass index
LVPWd: Left ventricular posterior wall end diastolic thickness
Lateral e′: Lateral mitral annular early diastolic peak velocities
Lateral E/e′: The ratio of early diastolic transmitral flow velocity to mitral annular velocity at the lateral wall
NT-proBNP: N-terminal pro-B-type natriuretic peptide
NYHA: New York Heart Association
Septal e′: Septal mitral annular early diastolic peak velocities
Septal E/e′: The ratio of the early diastolic transmitral filling velocity to the early diastolic septal tissue velocity
TAPSE: Tricuspid annular plane systolic excursion
TAPSE-S: Tricuspid annular plane systolic excursion velocity
