# Supplementary material for: The relationship between ambulatory arterial stiffness index and left ventricular diastolic dysfunction in HFpEF: a prospective observational study
Source: BMC Cardiovasc Disord. 2022 Jun 2;22:246. doi: 10.1186/s12872-022-02679-6 (PMC9161538; doi:10.1186/s12872-022-02679-6)
Supplement: Supplementary file 1 — Additional file 1. Table S1. Pearson correlations between clinical characteristics and right ventricular systolic function. Table S2. Logistic regression of AASI > 0.55 predicting mean E/e′ > 14. Figure S1. ROC curve for AASI to predict mean E/e′ > 14. [file 12872_2022_2679_MOESM1_ESM.docx]

Supplemental table 1. Pearson correlations between clinical characteristics and right ventricular systolic function.

|  | TAPSE | | TAPSE-S | |
| --- | --- | --- | --- | --- |
| parameters | *r* | *P* | *r* | *P* |
| Age | 0.191 | 0.054 | 0.110 | 0.271 |
| Height | 0.171 | 0.084 | -0.009 | 0.924 |
| Weight | 0.153 | 0.122 | 0.034 | 0.730 |
| BMI | 0.100 | 0.313 | 0.070 | 0.483 |
| Ave-sBP | 0.193 | 0.050 | 0.161 | 0.105 |
| Ave-dBP | -0.096 | 0.337 | -0.109 | 0.275 |
| AASI | 0.203 | 0.040 | 0.181 | 0.068 |

AASI: Ambulatory arterial stiffness index, Ave-sBP: Averaged systolic blood pressure, Ave-dBP: Averaged diastolic blood pressure, BMI: Body mass index, TAPSE: Tricuspid annular plane systolic excursion, TAPSE-S: Tricuspid annular plane systolic excursion velocity.

Supplemental table 2. Logistic regression of AASI>0.55 predicting mean E/e’ >14.

| Risk factors | Mean E/e’>14 | | | | | |
| --- | --- | --- | --- | --- | --- | --- |
|  | univariate | | | multivariate | | |
|  | OR | 95%CI | *P* | OR | 95%CI | *P* |
| AASI>0.55 | 2.817 | 1.224-6.481 | 0.015 | 2.457 | 1.030-5.860 | 0.043 |
| Age>65 | 2.071 | 0.850-5.047 | 0.109 |  |  |  |
| Ave-sBP >135 | 1.755 | 0.696-4.426 | 0.233 |  |  |  |
| BMI>25 | 0.717 | 0.305-1.687 | 0.446 |  |  |  |
| Female | 2.250 | 0.966-5.240 | 0.060 | 2.029 | 0.834-4.935 | 0.119 |
| Hypertension | 2.131 | 0.848-5.355 | 0.108 |  |  |  |
| Diabetes | 2.130 | 0.929-4.883 | 0.074 | 1.557 | 0.639-3.793 | 0.329 |
| CAD | 2.365 | 1.047-5.338 | 0.038 | 1.904 | 0.808-4.488 | 0.141 |
| COPD | 1.661 | 0.515-5.356 | 0.396 |  |  |  |

AASI: Ambulatory arterial stiffness index, Ave-sBP: Averaged systolic blood pressure, BMI: Body mass index, CAD: Coronary atherosclerotic heart disease, COPD: chronic obstructive pulmonary disease, Mean E/e’: Average septal-lateral E/e’ ratio.


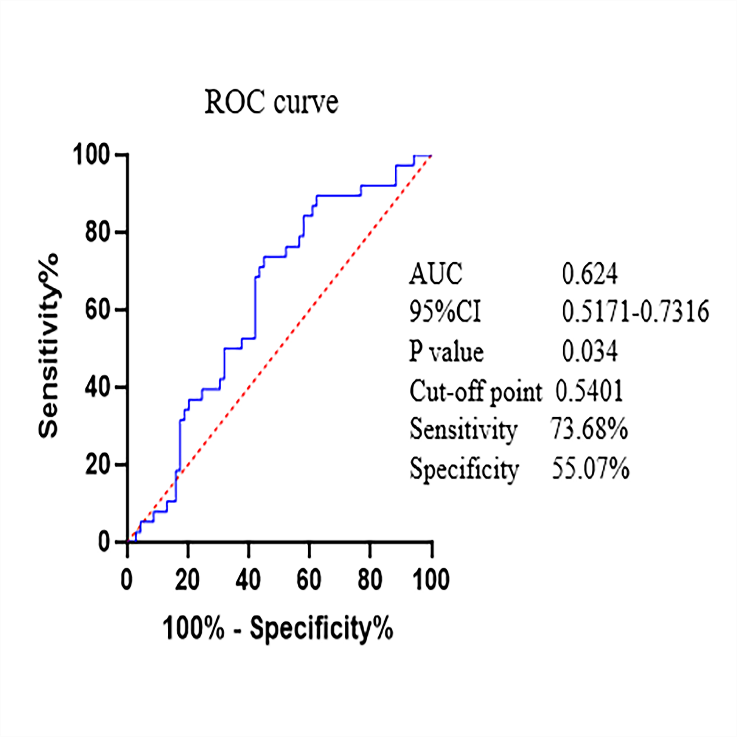


Supplemental figure 1. ROC curve for AASI to predict mean E/e’ >14.
